# Descriptive analysis of adverse drug reaction reports for hypersensitivity reactions stratified in relation to different beta-lactam antibiotics 

**DOI:** 10.5414/ALX02189E

**Published:** 2022-02-03

**Authors:** Diana Dubrall, Maike Schulz, Matthias Schmid, Bernhardt Sachs

**Affiliations:** 1 Institute of Medical Biometry, Informatics and Epidemiology, University Hospital Bonn, Venusberg-Campus 1, Bonn, Germany,; 2 Research Department, Federal Institute for Drugs and Medical Devices (BfArM), Bonn, Germany,; 3 Central Institute for the Provision of Health Care by Statutory Health Insurance Physicians in the Federal Republic of Germany, Berlin, Germany,; 4 Clinic for Dermatology and Allergology, University Hospital RWTH Aachen, Aachen, Germany

**Keywords:** hypersensitivity reaction, allergy, anaphylaxis, cutaneous reactions, beta-lactam antibiotics, penicillin, cephalosporin, adverse drug reactions, side effects, adverse drug reaction database analysis

## Abstract

β-lactam antibiotics (BLA) are commonly reported to induce hypersensitivity reactions. However, β-lactam antibiotic-stratified analyses are rare. In the presented study, β-lactam antibiotic associated hypersensitivity reactions were analyzed in the European adverse drug reaction (ADR) database. 923, 38, 222, and 99 hypersensitivity reports for penicillins and first-, second- and third-generation cephalosporins were reported. Differences with regard to demographical parameters, seriousness and types of hypersensitivity reactions, as well as in the number of hypersensitivity reports per outpatient prescriptions were observed between the different β-lactam antibiotics. The number of ADR reports classified as serious was higher for all generations of cephalosporins compared to penicillins. Additionally, anaphylactic reactions were more often reported for first- and second-generation cephalosporins compared to third-generation cephalosporins and penicillins, while bullous reactions were more often reported for first- and third-generation cephalosporins as opposed to second-generation cephalosporins and penicillins. The observed differences may be caused by differences between β-lactam antibiotics and their routes of administration (oral, intravenous), the patient populations, or the reporting of ADRs. Due to the methodological limitations of ADR database analysis, no conclusions can be drawn whether and to what extent the aforementioned factors influenced our results.

The views and interpretations set out by the authors do not necessarily reflect those of the Federal Institute for Drugs and Medical Devices. 

## Introduction 

Hypersensitivity reactions to drugs (adverse drug reactions (ADRs) type B) can be classified into allergic and nonallergic reactions (e.g., intolerance to analgesics) with respect to their pathophysiology [1]. Drug allergies refer to immunological hypersensitivity reactions, which may be mediated by IgE antibodies or T cells. According to studies, among the hypersensitivity reactions, the proportion of reported drug allergies is clearly higher than the proportion of medically confirmed drug allergies (~ 5 – 10%) [2, 3, 4, 5]. These discrepancies are based, among others, on 1) an overuse of the term “allergy”, 2) other factors causing the reaction (e.g., underlying infections) [2, 6], or 3) a decrease in detectability with increasing time interval to the reaction [2]. 

The estimated incidences and prevalences of hypersensitivity reactions to drugs described in different studies vary greatly. Differences in study design and inconsistently applied definitions and identifications of hypersensitivity reactions may account for some of these differences [7]. The estimated prevalence of dermatologist-verified allergic skin reactions was 3.6/1,000 for inpatients in a prospective study from France [8] and 1.8 – 4.2/1,000 inpatients for allergic skin reactions and systemic allergic reactions in studies from Korea and Singapore [9, 10]. In an Italian retrospective study of hospital records, drug allergy was suspected in 7.5% and 6.1% of adult and pediatric hospitalizations, respectively [11]. With respect to patients who consulted an allergologist due to suspected drug allergy, 26% were diagnosed with drug allergy in a prospective study from Spain [12]. Furthermore, in a study from Portugal, 7.8% of adults reported drug allergy [13]. In an ADR database analysis from Italy, ~ 1/10 (11.6%) of ADR reports referred to anaphylactic/anaphylactoid skin and systemic reactions [14]. 

In terms of their latency period between the application of the drug and the time to onset of the allergic reaction, hypersensitivity reactions can be classified as immediate-type (< 1 hour) or delayed-type (> 1 hour) reactions [1, 2, 6]. An immediate-type reaction typically manifests as urticaria, angioedema, or anaphylactic reaction. Maculopapular drug exanthema is the most common delayed-type reaction [2]. Stevens-Johnson syndrome (SJS) and toxic epidermal necrolysis (TEN) belong to the very rare occurring severe bullous delayed-type reactions [2, 6]. 

The estimated prevalences and incidences of anaphylactic reactions also vary depending on the study design and the definitions applied. In summary, for Western countries the estimated prevalences and incidences of anaphylactic reactions are between 8 and 50 per 100,000 person-years with a lifetime prevalence of 0.05 – 2% [4, 7, 15]. In these studies, penicillins were frequently described to induce IgE-mediated anaphylactic reactions, which were estimated to occur in ~ 0.015 – 0.04% of treated patients. In contrast, anaphylaxis is estimated to occur less frequently with the use of cephalosporins (0.0001 – 0.1%) [16]. 

For the bullous delayed-type reactions SJS and TEN, an incidence of 0.93 per 1 million population per year was determined in a study of the German Documentation Center for Severe Skin Reactions. There, a 3-fold higher relative risk (RR) of causing SJS/TEN was observed for cephalosporins compared to penicillins [17, 18]. 

Depending on the types of hypersensitivity reactions, various risk factors have been discussed in the literature. With respect to penicillin associated allergic reactions, underlying infections (e.g., Epstein-Barr virus (EBV) in ampicillin use), frequency of use (e.g., frequent intravenous administration in patients with cystic fibrosis), genetic factors, pre-existing personal or family drug allergy, and a multiple drug allergy syndrome, have been described [4, 7]. Whether these factors also apply to cephalosporins, carbapenems, and monobactams is unclear, as there is less robust data published, so far. Independently of the drugs, intravenous administration as well as asthmatic and atopic diseases have been discussed to be associated with severe courses of anaphylactic reactions [4, 15]. Comorbidities such as HIV infections and malignant diseases are, among others, reported to be associated with a more frequent occurrence of SJS/TEN [18, 19]. 

In summary, data, especially with regard to different β-lactam antibiotics, are still lacking. Thus, the first aim of this study was to determine the number of ADR reports referring to hypersensitivity reactions to β-lactam antibiotics in Germany. Second, these reports were then stratified by β-lactam antibiotics (including penicillins, cephalosporins) and analyzed with regard to their reported characteristics. Third, the number of hypersensitivity reports stratified by β-lactam antibiotics was related to the number of outpatient prescriptions. 

## Materials and methods 

### Reporting channels of ADR reports 

The reporting channels and reporting obligations are described elsewhere [20]. 

The analysis was performed in the European ADR database EudraVigilance of the European Medicines Agency (EMA) [21]. Here, drugs are coded in accordance with the EudraVigilance medicinal product dictionary (XEVMPD or Article 57 database) [22] and ADRs are coded in accordance with the MedDRA terminology [23]. The MedDRA terminology consists of five hierarchical levels of analysis which allows the reported ADRs to be analyzed in a summarized, aggregated, as well as a very specific manner. The levels of analysis used in the conducted study are the Preferred Term (PT) level, which describes the symptoms, diagnoses, and investigations, and the High Level Term (HLT) level, which corresponds to a grouping of the corresponding symptoms, diagnoses, and investigations based on their anatomy, pathology, and etiology. In this context, the MedDRA coding differs from the clinical coding of the clinical phenotypes (e.g., urticaria versus urticarial immediate-type reaction). Thus, for some symptoms the clinical phenotype cannot be assigned to immediate-type or delayed-type reactions. 

### Case identification 

**Figure 1.**
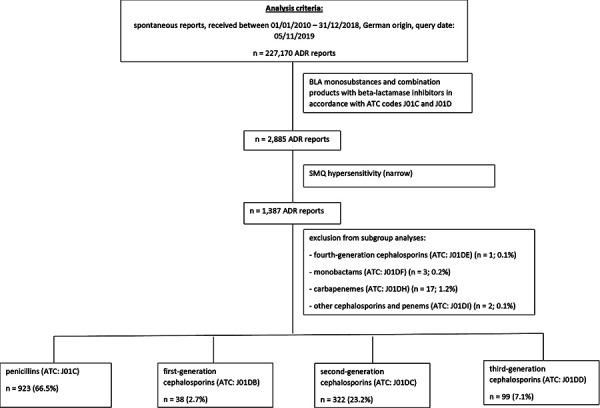
Flowchart.

A standardized MedDRA query (SMQ) [23] was used to identify all spontaneous reports of hypersensitivity reactions from Germany, received between January 01, 2010 and December 31, 2018, which reported a β-lactam antibiotic as a monosubstance or in combination with a β-lactamase inhibitor as suspected/interacting drug (n = 1,387, 48.1%) (Figure 1, Flowchart). The reported β-lactam antibiotics were assigned to the following subgroups of antibiotics based on current ATC coding: penicillins (J01C) (n = 923), first-generation cephalosporins (J01DB) (n = 38), second-generation cephalosporins (J01DC) (n = 322), third-generation cephalosporins (J01DD) (n = 99), fourth-generation cephalosporins (J01DE) (n = 1), monobactams (J01DF) (n = 3), carbapenems (J01DH) (n = 17), and other cephalosporins and penems (J01DI) (n = 2). Due to the small number of reports, subgroup analyses were not performed for fourth-generation cephalosporins, monobactams, carbapenems, and other cephalosporins and penems. 

### Strategy of analyses 

The hypersensitivity reports (n = 1,387) were analyzed with regard to demographic parameters and histories of the patients, seriousness criteria of reports, year of receive date, primary reporting sources, and most common types of hypersensitivity reactions reported. In addition, stratified analyses were performed with regard to 1) age groups, 2) β-lactam antibiotic subgroups (penicillins, first-, second-, and third-generation cephalosporins), 3) β-lactam antibiotic drugs, 4) anaphylactic reactions, and 5) bullous reactions. 

For the age-stratified analysis, the following age groups were used: 0 – 1 year, 2 – 3 years, 4 – 6 years, 7 – 12 years, 13 – 18 years, 19 – 65 years, and older than 65 years. The age classification of patients younger than 18 years corresponds to the age classification of the National Association of Statutory Health Insurance Physicians [24]. 

The drug-stratified analysis was performed for the five β-lactam antibiotics most frequently reported as suspected/interacting. In this analysis, the three most frequently reported indications and routes of administration, and the five most frequently reported types of hypersensitivity reactions, were determined. 

The analysis of the seriousness criteria of the hypersensitivity reports refers to the legal definition of a serious ADR in accordance with German Drug Law [25]. Reports are classified as serious if the reported ADR was life-threatening, resulted in hospitalization or prolongation thereof, or caused permanent disabilities, death, or congenital anomalies. In this regard, one hypersensitivity report may contain more than one seriousness criterion. It has to be noted that, the seriousness of an ADR report differs from the clinical severity of an ADR. 

The primary reporting source describes the person who compiled the report. This can be, among others, a physician, a pharmacist, or a patient. One hypersensitivity report may contain multiple primary reporting sources (e.g., physician and patient report independently). The analysis of the primary reporting sources shows the number of hypersensitivity reports with only one primary reporting source (e.g., physician). 

The types of hypersensitivity reactions most frequently reported were analyzed at the HLT level of MedDRA terminology [23]. For the stratified analysis of anaphylactic and bullous reactions, the hypersensitivity reports referring to the HLTs “anaphylactic and anaphylactoid responses” and “bullous conditions” were extracted. In the drug-stratified analysis, the reported indications were evaluated at the lower Preferred Term (PT) level (symptom level). 

In the stratified analysis of anaphylactic and bullous reactions, odds ratios (ORs) were calculated with Bonferroni-adjusted 95% confidence intervals (CI) for reports of anaphylactic or bullous reactions versus reports not reporting anaphylactic or bullous reactions. If the lower CI exceeds 1, the respective characteristic is more frequently reported for anaphylactic or bullous reactions. 

### Reporting rate per prescriptions 

The Research Institute for Ambulatory Health Care in Germany [26] provided the annual numbers of prescriptions for penicillins, first-, second-, and third-generation cephalosporins, and for the five β-lactam antibiotics most frequently reported as suspected/interacting in our analysis based on drug prescription data according to § 300 SGB V for the period 2010 – 2018. The provided data covers all prescriptions for statutory insured patients that were filled at a German pharmacy. Hence, prescriptions in hospitals and for members of the private health insurances are not included. In addition, no statements can be made whether the antibiotics prescribed were taken in accordance with the physician’s instructions. In order to calculate the reporting rate per 1,000,000 outpatient prescriptions, the number of hypersensitivity reports was divided by their number of outpatient prescriptions per year and multiplied with 1,000,000. For penicillins, first-, second-, and third-generation cephalosporins, the mean and median numbers of the reporting rates per year (2010-2018) with their standard deviations (STD), and interquartile ranges (IQR) were calculated. The QQ plots (quantile-quantile diagram) of the number of hypersensitivity reports per β-lactam antibiotic are presented in Supplement Figure 1. To assess whether there are differences in the numbers of hypersensitivity reports per 1,000,000 outpatient prescriptions between penicillins, first-, second-, and third-generation cephalosporins, the Kruskal-Wallis test was applied. Therefore, differences in medians and p-values were calculated using a Dunn test with Holm correction. Comparisons in which the p-value does not exceed the threshold of 0.05 were interpreted as differences in the median numbers of hypersensitivity reports. 

For the number of inpatient prescriptions, the published data of the anti-infective surveillance of the ADKA-if-DGI project were considered in order to contextualize the calculated reporting rates per 1,000,000 outpatient prescriptions [27]. Therefore, the documents with open access from the years 2012 – 2019 were used. In these documents, the exposure to anti-infectives is given as daily doses per 100 nursing days based on the calculated frequencies of more than 100 acute care hospitals from Germany. 

All analyses were performed using R statistical software (version 3.3.5). No individual case assessment of the reports was performed with respect to 1) the causal relationship between the reported hypersensitivity reaction and the reported suspected/interacting β-lactam antibiotic, 2) the accuracy of the diagnosis, and 3) the quality of documentation . No conclusions can be drawn whether the reported hypersensitivity reactions were investigated or confirmed by diagnostics. 

## Results 

### Reported characteristics in the overall data set and the *beta*-lactam antibiotic subgroups 

The average age of the patients in the total data set was 46.6 years (Table 1). Proportionally more reports referred to female (58.7%) than to male patients (38.9%). This was also seen for penicillins and second-generation cephalosporins. 

In 196 hypersensitivity reports (14.1%) of the total data set, a previous hypersensitivity reaction or allergy was recorded in the patient history. The proportion of patients with known hypersensitivity reactions or allergies was highest for the third-generation cephalosporins (17.2% (n = 17)). In contrast, prior hypersensitivities to other drugs were not recorded for any patient using first-generation cephalosporins. Cardiovascular diseases were more frequently described in patients who had used first- (10.5%) or third-generation cephalosporins (13.1%) than in patients who had taken second-generation cephalosporins (5.3%) or penicillins (3.3%). 

In the overall data set, approximately half (51.3%) of all reports (n = 1,387) were classified as serious, and approximately one-quarter (24.4%) reported a hospital admission or prolongation thereof. Death was noted in 2.9% of all hypersensitivity reports. Differences in the proportion of hypersensitivity reports classified as serious were seen between first- (84.2%), second- (70.5%), and third- (75.8%) generation cephalosporins compared to penicillins (40.7%). This was also observed for the criteria life-threatening, hospitalization, and death. 

The proportion of hypersensitivity reports originating from a single patient was 26.6% in the overall data set and was slightly lower for first- and third-generation cephalosporins than for the other two β-lactam antibiotic groups (Table 1). 

### Most common reported types of hypersensitivity reactions by *beta*-lactam antibiotic subgroups 

Rashes, eruptions, and exanthems were most frequently reported for penicillins (50.6%) and clearly less frequently for third- (38.4%), second- (25.8%), and first-generation (5.3%) cephalosporins. In contrast, anaphylactic/anaphylactoid reactions were most commonly reported for first- (52.6%) and second-generation (33.2%) cephalosporins, and clearly less often for third-generation cephalosporins (9.1%) and penicillins (4.7%). In each of the four β-lactam antibiotic subgroups, one specific β-lactam antibiotic dominated in the reports describing anaphylactic reactions. In these anaphylactic reaction reports to penicillins, first-, second-, and third-generation cephalosporins, 69.8% (n = 30) referred to amoxicillin (route of administration: oral), 95.0% (n = 19) referred to cefazolin (intravenous administration: 78.9%), 97.2% (n = 104) referred to cefuroxime (intravenous administration: 61.5%) and 77.8% (n = 7) referred to ceftriaxone (intravenous administration: 100.0%), respectively. 

Bullous reactions were most frequently reported for third-generation cephalosporins (14.1%), followed by first-generation cephalosporins (10.5%). In comparison, bullous reactions were reported less frequently for penicillins (4.8%) and second-generation cephalosporins (4.0%) 

Table 1 shows the characteristics reported in all hypersensitivity reports to β-lactam antibiotics and the stratified subgroups (penicillins, first-, second-, and third-generation cephalosporins). 

### Number of hypersensitivity reports and number of outpatient prescriptions per year 

The annual number of hypersensitivity reports was almost constant between 2010 and 2015 and increased for penicillins from 2016 and for second-generation cephalosporins from 2017 onwards. During the same period of time, a slight decrease of the annual number of hypersensitivity reports to first- and third-generation cephalosporins was observed. 

Similarly, the number of outpatient prescriptions was nearly constant for penicillins and second-generation cephalosporins during the period of analysis, while a slight decrease was observed for first- and third-generation cephalosporins. 

### Reporting rate per 1,000,000 outpatient prescriptions 

**Figure 2.**
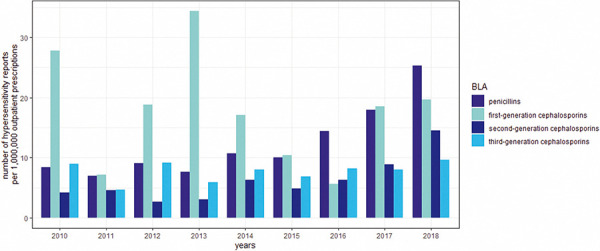
Annual number of hypersensitivity reports in relation to the number of 1,000,000 outpatient prescriptions (= reporting rate). In addition, the mean numbers of the reporting rates over the years were calculated. The mean numbers of hypersensitivity reports per 1,000,000 outpatient prescriptions per year were 12.3; 17.7; 6.2 and 7.7 for penicillins and first-, second-, and third-generation cephalosporins.

The *average* annual reporting rate of the number of hypersensitivity reports per 1,000,000 outpatient prescriptions was highest for first-generation cephalosporins (17.7 reports) (Figure 2) (Table 2.1, Table 2.2). The lowest reporting rate was calculated for second-generation cephalosporins (6.2 reports) (reporting rates for the most commonly reported drugs are shown in Table 4). The calculations confirmed the differences in the median numbers of the annual reporting rates per 1,000,000 outpatient prescriptions between first- and second-generation cephalosporins and between penicillins and second-generation cephalosporins. Stratified by sex, there was a higher reporting rate for penicillins and second-generation cephalosporins for female patients and for first- and third-generation cephalosporins for male patients. In relation to the number of outpatient prescriptions, anaphylactic/anaphylactoid reactions were reported more frequently with first- (9.8 reports) and second-generation cephalosporins (2.0 reports). With regard to bullous reactions, reporting rates were higher for first- (2.0 reports) and third-generation cephalosporins (1.1 reports) compared to the other β-lactam antibiotic subgroups. 

### Age-stratified analysis of hypersensitivity reports 

With rising age, the proportion of hypersensitivity reports classified as serious and life-threatening increased. Disabilities and/or death were only reported for adults (19 – 65, > 66) (Table 3). 

Rashes, eruptions, and exanthems were the hypersensitivity reactions most commonly reported in all age groups with decreasing frequencies with rising age (age groups > 6 years). Conversely, anaphylactic/anaphylactoid reactions were the second most frequently reported hypersensitivity reaction for patients older than 18 years and more frequently in the older compared to the younger age groups (< 18 years). 

Amoxicillin was the penicillin reported most frequently as suspected/interacting across all age groups and ranked first in all age groups except for children aged 2 – 3 years. Cefaclor was the most commonly reported cephalosporin up to the age group of 13 – 18 years (ranked second expect for the age group of 2 – 3 years (ranked first)), and cefuroxime was the second most frequently reported cephalosporin and ranked second from the age group of 13 – 18 years onwards. 

### Drug-stratified analysis of hypersensitivity reports 

Amoxicillin (52.5%) was the β-lactam antibiotic most commonly reported as suspected/interacting followed by cefuroxime (n = 267, 19.3%), ampicillin/sulbactam (n = 80, 5.8%), cefaclor (n = 55, 4.0%), and phenoxymethylpenicillin (n = 52, 3.7%) (Table 4). Rashes, eruptions, and exanthems were the most common reported types of hypersensitivity reactions for four out of the five β-lactam antibiotics (amoxicillin: 56.9%; ampicillin/sulbactam: 52.5%; cefaclor: 49.1%; phenoxymethylpenicillin: 46.2%). In contrast, anaphylactic/anaphylactoid reactions (39.0%) were most commonly reported for cefuroxime. Furthermore, the seriousness criteria death and life-threatening were coded more frequently for cefuroxime compared to the other β-lactam antibiotics. The most common reported indication for cefuroxime was antibiotic prophylaxis. Cefuroxime (32.4%) and ampicillin/sulbactam (29.3%) were more often administered intravenously compared to the other four β-lactam antibiotics. In addition, with 69.6% (n = 64) the proportion of anaphylactic/anaphylactoid reactions was clearly higher in the reports with intravenous administration of cefuroxime (n = 92) compared to the reports that did not explicitly mention intravenous administration (22.8%, n = 40). 

The highest reporting rate was calculated for ampicillin/sulbactam with 296.6 hypersensitivity reports per 1,000,000 outpatient prescriptions. Further subdivision into the active ingredient ampicillin and the combination ampicillin and sulbactam resulted in 24.0 and 7,677.1 hypersensitivity reports per 1,000,000 outpatient prescriptions, respectively. In an individual case assessment of reports for the drug combination ampicillin and sulbactam, 16/74 reports were assumed to be related to drug exposure in an inpatient setting (inpatient prescriptions are not included in the denominator of outpatient prescriptions). In the remaining 58 reports, there was no evidence of an inpatient prescription, nevertheless it cannot be excluded with certainty. 

The second highest reporting rate was calculated for amoxicillin with 13.8 hypersensitivity reports, followed by cefuroxime (6.9 hypersensitivity reports), cefaclor (3.8 hypersensitivity reports), and phenoxymethylpenicillin (3.1 hypersensitivity reports). With regard to the age groups, an increase of the reporting rates with rising age was observed for ampicillin/sulbactam. Similarly, for amoxicillin and cefuroxime the reporting rates were slightly higher for the age groups older than 18 years than for the younger age groups. Conversely, for cefaclor the reporting rate was slightly higher in the younger age groups (0 – 12 years) compared to the older age groups (≥ 13 years). The reporting rates for phenoxymethylpenicillin varied between the age groups. 

### Stratified analysis according to anaphylactic and bullous reactions 

Patients who experienced anaphylactic reactions were more likely diagnosed with hypertension (OR 2.5 [1.0 – 6.1]), cardiovascular diseases (OR 2.9 [0.9 – 9.5]), asthma (OR 4.7 [0.9 – 23.1]), chronic obstructive pulmonary diseases (COPD) (OR 4.8 [0.9 – 25.7]), and diabetes (OR 2.5 [0.7 – 8.4] (Table 5)). Likewise, hypertension (OR 2.1 [0.7 – 5.9]), cardiovascular diseases (OR 3.0 [0.9 – 10.0]), COPD (OR 3.5 [0.6 – 19.1]), and diabetes (OR 2.5 [0.7 – 9.0]) were reported more frequently for bullous reactions considering the effect estimates (OR). However, in both stratified analyses no clear associations with anaphylactic and bullous reactions were seen for patients with previous hypersensitivity reactions and allergies. Both reactions were more frequently associated with the seriousness criteria serious, death, life-threatening, and hospitalization. 

Compared with reports not reporting anaphylactic reactions, cefazolin (OR 15.5 [2.7 – 88.1]) and cefuroxime (OR 8.6 [5.0 – 14.7]) were more frequently associated with anaphylactic reactions. 

In contrast, ceftazidime (OR 26.1 [3.3 – 207.9]) and piperacillin (OR 7.6 [2.2 – 26.3]) were more frequently suspected in reports of bullous reactions compared to reports not reporting bullous reactions. 

## Discussion 

To our knowledge, this is the first analysis of β-lactam antibiotic associated hypersensitivity reactions stratified by types of β-lactam antibiotics performed for Germany in the European ADR database. In our analysis, differences between the β-lactam antibiotics were observed with regard to the seriousness of the hypersensitivity reports and the most frequently reported types of hypersensitivity reactions. 

### Demographic parameters 

According to the literature, the proportion of female patients is higher than the proportion of male patients with regard to 1) self-reported hypersensitivity reactions in electronic health records (EHR) [28], 2) consultations of allergists for suspected β-lactam antibiotic allergies [12], and 3) hospital admissions due to hypersensitivity reactions [29]. In addition, allergies to penicillins and cephalosporins were recorded more frequently for females than for males in EHR data [6, 30, 31]. In our analysis, the proportion of female compared to male patients was higher for the total data set and for reports referring to penicillins and second-generation cephalosporins. However, this was not the case for first- and third-generation cephalosporins. The higher proportion of reports for females in our analysis (total data set) could, among others, also be related to 1) a more frequent reporting of symptoms and ADRs [32, 33], 2) more frequent visits to physicians [34] and consequently a more frequent use of drugs [35], and 3) a more frequent visit of healthcare facilities and emergency departments by females compared to males [36, 37, 38]. Furthermore, differences in drug use (including indications) between men and women could account for differences in β-lactam antibiotic-specific exposures. Other studies [39] as well as the outpatient prescription data from Germany [26] showed a more frequent prescription of antibiotics, especially cephalosporins, to women compared to men. Thus, in relation to the outpatient prescriptions, higher reporting rates were observed for first- and third-generation cephalosporins for male compared to female patients in our analysis. This observation differs from the literature cited above. However, the studies mentioned above did not consider frequencies of drug exposure or stratifications by cephalosporins. 

In our analysis, the number of hypersensitivity reports increased with rising age up to the age of 65 years. A more frequent occurrence of hypersensitivity reactions in adults than in children, as well as a more frequent documentation in EHR data with rising age [30] has already been described in the literature. This can be explained by a higher cumulative exposure from recurrent drug use [4, 16] and a higher number of patients with chronic diseases and polypharmacy [7, 40, 41] with rising age. Regarding the less frequent occurrence of hypersensitivity reactions in children [4] and the very elderly (> 80) [42], differences in immune responses have been discussed. Further on, other studies suggested that underlying infections as differential diagnoses should be considered more frequently in children [43]. 

### Seriousness of hypersensitivity reports in accordance with the legal definition 

The higher proportion of hypersensitivity reports classified as serious with rising age might also be associated with the more frequent use of multiple drugs and the more frequent presence of chronic diseases [40, 41, 44]. The increase of serious ADR reports with rising age has already been reported for Germany without specifying the reaction [45]. In our analysis, death was only coded for reports referring to patients older than 18 years. It has to be noted though that without an individual case assessment of these reports, no firm conclusions can be drawn whether the hypersensitivity reaction itself or other causes, such as underlying diseases, are responsible for the fatal outcome. 

In our analysis, hypersensitivity reports to cephalosporins were clearly more often classified as serious, fatal, life-threatening, or reported hospitalization or prolongation thereof compared to penicillins. To date, we are not aware of a direct comparison of the seriousness or severity of hypersensitivity reactions stratified by β-lactam antibiotic. Only specific analyses referring to severe types of hypersensitivity reactions such as anaphylactic reactions, can be found in the literature. 

### Anaphylactic reactions

In relative numbers, anaphylactic reactions were reported more frequently for cephalosporins compared to penicillins in our analysis. In other studies, anaphylactic reactions occurred more frequently in association with penicillins than with cephalosporins [46, 47, 48]. In a recent study of the German Anaphylaxis Registry, the use of a penicillin was reported in 41% and the use of a cephalosporin in 32% of the analyzed anaphylactic reactions [49]. In contrast, in a study from the Allergy Center in Würzburg, 84.3% of confirmed immediate-type reactions were caused by cephalosporins [50]. Consistent with our analysis, the most commonly reported cephalosporins in the study from Würzburg were cefuroxime, cefazolin, and ceftriaxone [50]. Without consideration of the exact outpatient and inpatient prescription figures, the frequency of anaphylactic reactions stratified by β-lactam antibiotic can only be speculated. With regard to outpatient prescriptions, penicillins are prescribed clearly more often than cephalosporins in Germany [51] as well as in other European countries [52, 53, 54]. For inpatient prescriptions, this is not the case for each of the European countries, but penicillin is more frequently prescribed, summarized for all countries of the EU [54]. For more than 100 acute care hospitals in Germany, the summarized inpatient prescription was higher for penicillins than for cephalosporins since 2015. Although anaphylactic reactions in absolute terms are observed more frequently for penicillins than for cephalosporins, they might occur relatively less frequently than with cephalosporins if the number of prescriptions (outpatient + inpatient) is taken into account. 

The route of administration (e.g., oral or intravenous) of the respective β-lactam antibiotic could also influence the occurrence of anaphylaxis. The intravenous route of administration has been reported in literature as a risk factor for the occurrence of anaphylactic reactions [46, 49, 55]. Amoxicillin, the most frequently reported penicillin in the anaphylactic reaction reports in our analysis, is used orally, whereas the most frequently reported first- (cefazolin), second- (cefuroxime), and third- (ceftriaxone) generation cephalosporins were administered intravenously in a high proportion of our reports. In the study from Würzburg [50] and a Turkish study in children [56], ~ 50% and 81.8% of confirmed immediate-type reactions were related to intravenous administration of a β-lactam antibiotic and a cephalosporin, respectively. 

Cefazolin and cefuroxime were also more frequently associated with anaphylactic reactions in our stratified analysis. Since cefuroxime was frequently used for perioperative antibiotic prophylaxis, other drugs, such as drugs used to induce anesthesia, might also be responsible. However, a Spanish study concluded that cephalosporins were the drugs most commonly associated with hypersensitivity reactions during anesthesia compared to other drugs used for induction of anesthesia [57]. 

In addition, some comorbidities, e.g., cardiovascular diseases, have also mentioned in the literature as risk factors for the occurrence of anaphylactic reactions [16, 49, 58]. In our analysis, cardiovascular diseases were more frequently described for patients who had used cephalosporins and who suffered from anaphylactic reactions. 

### Bullous reactions 

Differences between the β-lactam antibiotics were also seen with regard to bullous reactions. Our data showed a higher proportion of reports for third- and first-generation cephalosporins than for penicillins and second-generation cephalosporins. In particular, ceftazidime (third-generation cephalosporin) was clearly more frequently associated with bullous reactions. Despite proportionally fewer reported bullous reactions to penicillins, piperacillin was more commonly suspected in reports of bullous reactions in the stratified analysis. The EuroSCAR study of hospitalizations due to SJS/TEN showed a slightly higher proportion of cephalosporin-exposed patients than penicillin-exposed patients, as well as a higher risk of SJS/TEN for cephalosporins compared with their respective control groups [18]. Considering the differences between outpatient and inpatient prescriptions, a more frequent occurrence of SJS/TEN could be suspected for cephalosporins compared to penicillins. Whether the frequencies of SJS/TEN differed between different generations of cephalosporins cannot be conclusively assessed in our analysis. 

### Frequency of hypersensitivity reports in relation to the number of outpatient prescriptions 

In relation to the number of outpatient prescriptions, the calculated reporting rate was higher for first-generation cephalosporins, followed by penicillins and third- and second-generation cephalosporins. Penicillins have been reported as the most commonly suspected drugs of hypersensitivity reactions in several studies [7, 28, 30]. However, these studies rarely considered the frequencies of their exposure. In addition, differences in prescribing behaviors or individual predispositions, such as genetic differences, may also complicate the comparability of data from other countries to Germany. In our calculation, it has to be noted that the number of prescriptions only reflects the *outpatient* prescriptions for statutory insured patients. However, the number of ADR reports can include all reported hypersensitivity reactions, regardless of whether the suspected β-lactam antibiotic was prescribed in an outpatient or inpatient setting. Since 2015, penicillins were prescribed more frequently than cephalosporins in inpatient settings [27]. Among these, inpatient frequencies of use were higher for first- and second-generation cephalosporins than for third- and fourth-generation cephalosporins (since 2012). However, it cannot be assessed whether this data acquisition is representative for the inpatient prescriptions in all German hospitals. In addition, inpatient prescriptions could differ greatly between the different β-lactam antibiotics, severely limiting the comparability of the calculated reporting rates per 1,000,000 outpatient prescriptions. By definition, the coding “hospitalization” covers all ADRs that led to hospitalization (i.e., presumably prescribed in the outpatient setting) or prolonged hospitalization (i.e., presumably prescribed in the inpatient setting). The higher proportion of reports with hospitalizations related to cephalosporins in our analysis could therefore also indicate more reports of ADRs prolonging a hospital stay, and suggesting a prescription in the inpatient setting. This would lead to an overestimation of the reporting rates to cephalosporins compared to penicillins. Unfortunately, this cannot be examined in our analysis. 

Ampicillin/sulbactam was the second most commonly prescribed penicillin in the inpatient setting since 2011 [27]. Extrapolated to our analysis, this may have generated the high reporting rate per 1,000,000 outpatient prescriptions, as the number of inpatient prescriptions is missing in the denominator (thereby increasing the value of the quotient). The number of inpatient prescriptions for ampicillin (single ingredient) was clearly lower (data available since 2016) than for the combination ampicillin/sulbactam and amoxicillin, but slightly higher than for phenoxymethylpenicillin. 

Cefuroxime was the most frequently prescribed cephalosporin in inpatient prescriptions since 2007 and was prescribed clearly more often than cefaclor suggesting that the reporting rate for cefuroxime is also overestimated. This is supported by the high proportion of reports with the indication perioperative prophylaxis, which are probably due to an inpatient prescription. 

### Advantages and disadvantages of our analysis and of analyses in spontaneous reporting systems 

The advantages of analyses in ADR databases are the identification of very rare ADRs as well as ADRs that occur during long-term use of a drug or in certain clinical situations (e.g., comorbidities) or interactions with other drugs [20]. In addition, ADRs of vulnerable patients, who are often not included in clinical trials, such as very young/old patients, are covered. 

Limitations of the spontaneous reporting systems include the unknown amount of under-reporting and the lack of accurate patient-specific exposure data. It is estimated that only ~ 5 – 10% of all ADRs are reported [59], and the frequency of reporting may additionally depend on other factors (e.g., seriousness) [60]. Therefore, based on such analyses, ADR incidences cannot be calculated. An approximation may be generated by the calculation of reporting rates (number of reports/number of prescriptions). However, our analysis is limited by relating the exposure only to the outpatient prescriptions since data for inpatient prescriptions were not available. However, the ADRs reported could also be related to inpatient prescriptions. In addition, the number of inpatient prescriptions appears to vary widely between the β-lactam antibiotics [27], severely limiting the comparability of reporting rates per outpatient prescriptions stratified by β-lactam antibiotic. 

Due to the high number of reports, we did not perform an individual case assessment. All reports included in the presented analysis are suspected cases of hypersensitivity reactions. However, it has to be noted that 1) the causal relationship between the intake of the drug and the occurrence of the hypersensitivity reaction, 2) the correctness of the reported diagnosis (e.g., differential diagnosis of infection), 3) the proportion of diagnostically examined and confirmed hypersensitivity reactions, 4) the occurrence of immediate- or delayed-type reaction, and 5) cross-reactions, have not be assessed. 

## Conclusion 

Our analysis showed differences between the β-lactam antibiotics with regard to the reported types of hypersensitivity reactions (including anaphylactic reactions) and seriousness of hypersensitivity reports. In particular, intravenous administration of cephalosporins appears to be associated with a more frequent occurrence of anaphylactic reactions. Whether anaphylactic as well as bullous reactions occur more frequently with cephalosporins than with penicillins cannot be conclusively assessed from our data due to the limitations mentioned above (e.g., lack of exact inpatient prescriptions). Therefore, further studies are necessary to investigate the observed differences from our analysis. 

## Acknowledgment 

We thank the Department of Pharmacovigilance at the Federal Institute for Drugs and Medical Devices (BfArM) for their support. We also thank our colleagues at the Institute of Medical Biometry, Informatics and Epidemiology (IMBIE) at the University Hospital Bonn for their support. 

## Funding 

The Project is funded by the Federal Institute for Drugs and Medical Devices (BfArM) own resources and the Institute for Medical Biometry, Informatics, and Epidemiology (IMBIE), University Hospital of Bonn (V-16703/68502/2016-2020).

## Conflict of interest 

No conflict of interest.

**Table 1.** Characteristics of the total data set and the β-lactam antibiotic subgroups penicillins, first-, second- and third-generation cephalosporins.

	**Total data set ** **(n = 1,387)**	**Penicillins ** **(n = 923, 66.5%)**	**First-generation cephalosporins (n = 38, 2.7%)**	**Second-generation cephalosporins n = 322, 23.2%)**	**Third-generation cephalosporins (n = 99, 7.1%)**
Demographic parameters of the patients					
Average age of patients (median) [in years]^1^	46.6 (50)	46.8 (50)	43.3 (37)	47.2 (53)	41.5 (42)
Female	58.7% (n = 814)	60.1% (n = 555)	42.1% (n = 16)	59.3% (n = 191)	49.5% (n = 49)
Male	38.9% (n = 539)	37.9% (n = 350)	52.6% (n = 20)	36.3% (n = 117)	50.5% (n = 50)
Sex unknown	2.5% (n = 34)	2.0% (n = 18)	5.3% (n = 2)	4.3% (n = 14)	0.0% (n = 0)
Patient history					
Hypertension	8.9% (n = 123)	8.3% (n = 77)	13.2% (n = 5)	9.6% (n = 31)	11.1 (n = 11)
Cardiovascular diseases	4.7% (n = 65)	3.3% (n =30)	10.5% (n = 4)	5.3% (n =1 7)	13.1% (n =13)
Diabetes	4.7% (n = 65)	3.7% (n = 34)	5.3% (n = 2)	5.9% (n = 19)	8.1% (n = 8)
Asthma	2.2% (n = 30)	1.5% (n = 14)	2.6% (n = 1)	3.7% (n = 12)	2.0% (n = 2)
COPD^2^	1.9% (n = 27)	0.8% (n = 7)	5.3% (n = 2)	3.7% (n = 12)	5.1% (n = 5)
Hypersensitivities/allergies^3^	14.1% (n = 196)	13.1% (n = 121)	13.2% (n = 5)	15.5% (n = 50)	17.2% (n = 17)
Smoker	4.7% (n = 65)	4.4% (n = 41)	7.9% (n = 3)	5.6% (n = 18)	3.0% (n = 3)
Seriousness of hypersensitivity reports^4^					
Serious	51.3% (n = 712)	40.7% (n = 376)	84.2% (n = 32)	70.5% (n = 227)	75.8% (n = 75)
Death	2.9% (n = 40)	1.5% (n = 14)	2.6% (n = 1)	4.0% (n = 13)	13.1% (n = 13)
Life-threatening	11.4% (n = 158)	4.9% (n = 45)	47.4% (n = 18)	25.8% (n = 83)	14.1% (n = 14)
Hospitalization	24.4% (n = 339)	19.7% (n = 182)	34.2% (n = 13)	35.1% (n = 113)	34.3% (n = 34)
Disability	1.4% (n = 20)	1.4% (n = 13)	0.0% (n = 0)	1.6% (n = 5)	2.0% (n = 2)
Primary reporting source of the hypersensitivity reports^5^					
Physician	43.0% (n = 596)	45.0% (n = 415)	60.5% (n = 23)	33.2% (n = 107)	46.5% (n = 46)
Pharmacist	17.6% (n = 244)	15.7% (n = 145)	15.8% (n = 6)	22.4% (n = 72)	21.2% (n = 21)
Patient	26.6% (n = 369)	29.9% (n = 276)	10.5% (n = 4)	23.9% (n = 77)	12.1% (n = 12)
The three most frequently reported active ingredients					
1.	52.5% amoxicillin (n = 728)	78.9% amoxicillin (n = 728)	76.3% cefazolin (n = 29)	82.9% cefuroxime (n = 267)	44.4% ceftriaxone (n = 44)
2.	19.3% cefuroxime (n = 267)	8.7% ampicillin/sulbactam (n = 80)	13.2% cefadroxil (n = 5)	17.1% cefaclor (n = 55)	17.2% cefpodoxime (n = 17)
3.	5.8% ampicillin/sulbactam (n = 80)	5.6% phenoxymethylpenicillin (n = 52)	10.5% cefalexin (n = 4)		14.1% cefotaxime (n = 14)
The five most commonly reported types of hypersensitivity reactions (HLT level)^6^					
1.	42.7% rashes, eruptions, and exanthems ANE (n = 592)	50.6% rashes, eruptions, and exanthems ANE (n = 467)	52.6% anaphylactic and anaphylactoid reactions (n = 20)	33.2% anaphylactic and anaphylactoid reactions (n = 107)	38.4% rashes, eruptions, and exanthems ANE (n = 38)
2.	13.1% anaphylactic and anaphylactoid reactions (n = 182)	11.8% urticarial manifestations (n = 109)	13.2% allergic diseases ANE (n = 5)	25.8% rashes, eruptions, and exanthems ANE (n = 83)	14.1% bullous reactions (n = 14)
3.	11.5% urticarial manifestations (n = 160)	11.3% allergic diseases ANE (n = 104)	10.5% bronchospasm and obstruction (n = 4).	11.8% urticarial manifestations (n = 38)	13.1% allergic diseases ANE (n = 13)
4.	11.2% allergic diseases ANE (n = 155)	6.4% dermatitis caused by specific agent (n = 59)	10.5% bullous reactions (n = 4)	10.6% allergic diseases ANE (n = 34)	9.1% anaphylactic and anaphylactoid reactions (n = 9)
5.	5.8% dermatitis and eczema (n = 80)	6.4% pruritus ANE (n = 59)	10.5% urticarial manifestations (n = 4)	5.9% circulatory collapse and shock (n = 19) 5.9% dermatitis and eczema (n = 19)	8.1% angioedema (n = 8)

HLT = High Level Term (analysis level of MedDRA terminology), ANE = not classified elsewhere. ANE is used in MedDRA terminology to record groupings that do not fit into other higher-level codes of the respective system organ class (SOC). The SOC describes the organ in which the reaction occurs. ^1^In 16.1% (n = 223) of hypersensitivity reports the age of the patient was unknown. ^2^Chronic obstructive pulmonary disease (COPD). ^3^Reported hypersensitivities and allergies in the patient history are pooled together since a clear separation into hypersensitivities and allergies is not possible due to the non-differentiating coding. ^4^The seriousness of the ADR report is based on the legal definition of the German Drug Law [30]. An ADR report is classified as serious if the reported ADR was serious or life-threatening, resulted in hospitalization or prolongation thereof, led to death, or permanent disability or a congenital anomaly. ^5^Only reports referring to a single primary reporting source are shown. For example, reports which were reported by a physician and a patient, are not considered in this analysis. ^6^Shown are the five most commonly reported types of hypersensitivity reactions at the HLT level of the MedDRA terminology [28]. One hypersensitivity report may contain multiple types of hypersensitivity reactions. These may be assigned to different higher-level codes. As a result, the total number of types of hypersensitivity reactions coded at the HLT level shown, exceeds the total number of hypersensitivity reports.

**Table 2.1.** Mean and median numbers of hypersensitivity reports, the number of hypersensitivity reports for female and male patients, and for anaphylactic and bullous reactions in relation to 1,000,000 outpatient prescriptions stratified by β-lactam antibiotics. Bullous reactions were subdivided into SJS and TEN.

**Per 1,000,000 outpatient prescriptions**	**Penicillins ** **(n = 923, 66.5%)**	**First-generation cephalosporins (n = 38, 2.7%)**	**Second-generation cephalosporins (n = 322, 23.2%)**	**Third-generation cephalosporins (n = 99, 7.1%)**
Mean number of hypersensitivity reports (± STD) (2010 – 2018)	12.3 (± 6.0)	17.7 (± 9.3)	6-.2 (± 3.6)	7.7 (± 1.6)
Median number of hypersensitivity reports (IQR) (2010 – 2018)	10.0 (8.4 – 14.4)	18.5 (10.4 – 19.7)	4.9 (4.2 – 6.3)	8.0 (6.9 – 9.0)
Number of hypersensitivity reports for females	13.3	13.7	6.3	6.0
Number of hypersensitivity reports for males	10.0	20.1	5.1	8.8
HLT anaphylactic/anaphylactoid reactions	0.6^1^	9.8^2^	2.0^3^	0.7^4^
HLT bullous reactions	0.6	2.0	0.2	1.1
PT Stevens Johnson Syndrome (SJS)	0.2	0.0	0.0	0.5
PT Toxic epidermal necrolysis (TEN)	0.2	1.5	0.1	0.5

HLT = High Level Term (analysis level of MedDRA terminology); PT = Preferred Term (analysis level of MedDRA terminology). ^1^69.8% (n = 30) of reports amoxicillin (penicillin, type of route of administration: oral) reported as suspected. ^2^95.0% (n = 19) of reports cefazolin (first-generation cephalosporins, intravenous use: 78.9%) reported as suspected. ^3^97.2% (n = 104) of reports cefuroxime (second-generation cephalosporins, intravenous use: 61.5%) reported as suspected. ^4^77.8% (n = 7) of reports ceftriaxone (third-generation cephalosporins, intravenous use: 100.0%) reported as suspected.

**Table 2.2.** Results of calculations of differences in the median numbers of hypersensitivity reports per 1,000,000 outpatient prescriptions between penicillins, first-, second-, and third-generation cephalosporins.

**Comparative analyses**	**Medians of the comparison groups**	**p-values**
Penicillins – first-generation cephalosporins	10.0 – 18.5	0.437
Penicillins – second-generation cephalosporins	10.0 – 4.9	0.028
Penicillins – third-generation cephalosporins	10.0 – 8.0	0.368
First-generation cephalosporins – third-generation cephalosporins	18.5 – 8.0	0.080
Second-generation cephalosporins – first-generation cephalosporins	4.9 – 18.5	0.002
Second generation cephalosporins – third-generation cephalosporins	4.9 – 8.0	0.437

**Table 3.** Age-stratified analysis of characteristics reported in the hypersensitivity reports to β-lactam antibiotics.

	**Number of reports age group 0 – 1 year (n = 22)** *^1^*	**Number of reports age group 2 – 3 years (n = 25)** *^1^*	**Number of reports age group 4 – 6 years (n = 39)1**	**Number of reports age group 7 – 12 years (n = 52)** *^1^*	**Number of reports age group 13 – 18 years (n = 41)** *^1^*	**Number of reports age group 19 – 65 years (n = 710)** *^1^*	**Number of reports age group over 65 years (n = 275)** *^1^*

Sex of the patients							
Female	40.9% (n = 9)	40.0% (n = 10)	51.3% (n = 20)	46.2% (n = 24)	61.0% (n = 25)	62.0% (n = 440)	56.7% (n = 156)
Male	50.0% (n = 11)	60.0% (n = 15)	48.7% (n = 19)	53.8% (n = 28)	34.1% (n = 14)	37.0% (n = 263)	42.9% (n = 118)
Unknown	9.1% (n = 2)	0.0% (n = 0)	0.0% (n = 0)	0.0% (n = 0)	4.9% (n = 2)	1.0% (n = 7)	0.4% (n = 1)
Patient histories							
Hypertension	–	–	–	–	–	8.2% (n = 58)	20.7% (n = 57)
Cardiovascular diseases	–	–	2.6% (n = 1)	–	–	2.7% (n = 19)	15.6% (n = 43)
Diabetes	–	–	–	–	–	3.7% (n = 26)	13.5% (n = 37)
Asthma	–	–	–	1.9% (n = 1)	2.4% (n = 1)	3.0% (n = 21)	1.8% (n = 5)
COPD^2^	–	–	–	–	–	2.0% (n = 14)	4.7% (n = 13)
Hypersensitivities/allergies^3^	4.5% (n = 1)	4.0% (n = 1)	5.2% (n = 2)	15.4% (n = 8)	14.6% (n = 6)	15.5% (n = 110)	16.7% (n = 46)
Seriousness of hypersensitivity reports^4^							
Serious	27.3% (n = 6)	40.0% (n = 10)	41.0 (n = 16)	40.4% (n = 21)	48.8% (n = 20)	49.2% (n = 349)	63.3% (n = 174)
Death	0.0% (n = 0)	0.0% (n = 0)	0.0% (n = 0)	0.0% (n = 0)	0.0% (n = 0)	2.8% (n = 20)	5.5% (n = 15)
Life-threatening	4.5% (n = 1)	0.0% (n = 0)	5.1% (n = 2)	7.7% (n = 4)	7.3% (n = 3)	12.7% (n = 90)	16.0% (n = 44)
Hospitalization	13.6% (n = 3)	20.0% (n = 5)	23.1% (n = 9)	15.4% (n = 8)	22.0% (n = 9)	25.4% (n = 180)	31.3% (n = 86)
Disabling	0.0% (n = 0)	0.0% (n = 0)	0.0% (n = 0)	0.0% (n = 0)	0.0% (n = 0)	1.1% (n = 8)	3.3% (n = 9)
Number of reports per antibiotic subgroup							
Penicillins	6.,6% (n = 14)	44.0% (n = 11)	56.4% (n = 22)	57.7% (n = 30)	58.5% (n = 24)	70.1% (n = 498)	65.8% (n = 181)
First-generation cephalosporins	4.5% (n = 1)	4.0% (n = 1)	3.8% (n = 1)	3.8% (n = 2)	2.4% (n = 1)	2.0% (n = 14)	3.3% (n = 9)
Second-generation cephalosporins	22.7% (n = 5)	48.0% (n = 12)	28.2% (n = 11)	23.1% (n = 12)	22.0% (n = 9)	21.5% (n = 153)	22.5% (n = 62)
Third-generation cephalosporins	13.6% (n = 3)	4.0% (n = 1)	15.4% (n = 6)	19.2% (n = 10)	12.2% (n = 5)	6.2% (n = 44)	7.3% (n = 20)
The five most common β-lactam antibiotics reported as suspected/interacting^5^							
1.	50.0% amoxicillin (n = 11)	40.0% cefaclor (n = 10)	46.2% amoxicillin (n = 18)	38.5% amoxicillin (n = 20)	51.2% amoxicillin (n = 21)	56.8% amoxicillin (n = 403)	47.3% amoxicillin (n = 130)
2.	22.7% cefaclor (n = 5)	36.0% amoxicillin (n = 9)	20.5% cefaclor (n = 8)	15.4% cefaclor (n = 8)	19.5% cefuroxime (n = 8)	20.4% cefuroxime (n = 145)	21.8% cefuroxime (n = 60)
3.	9.1% ampicillin/sulbactam (n = 2)	8.0% phenoxymethyl penicillin (n = 2)	7.7% phenoxymethyl penicillin (n = 3).	13.5% phenoxymethyl penicillin (n = 7)	7.3% cefotaxime (n = 3)	6.2% ampicillin/ sulbactam (n = 44)	8.4% ampicillin/sulbactam (n = 23)
4.		8.0% cefuroxime (n = 2)	7.7% cefuroxime (n = 3)	11.5% ceftriaxone (n = 6)	4.9% meropenem (n = 2)	3.1% phenoxymethylpenicillin (n = 22)	5.1% ceftriaxone (n = 14)
5.						2.5% ceftriaxone (n = 18)	4.7% piperacillin (n = 13)
The five most common reported types of hypersensitivity reactions (HLT level)^6^							
1.	54.5% rashes, eruptions and exanthems (n = 12)^7^	52.0% rashes, eruptions and exanthems (n = 13)^7^	56.4% rashes, eruptions and exanthems (n = 22)^7^	67.3% rashes, eruptions and exanthems (n = 35)^7^	41.5% rashes, eruptions and exanthems (n = 20)^7^	38.0% rashes, eruptions and exanthems (n = 337)^7^	28.7% rashes, eruptions and exanthems (n = 108)^7^
2.	13.6% dermatitis triggered by specific agent (n = 3)	32.0% urticaria (n = 8)	28.2% urticaria (n = 11)	19.2% urticaria (n = 10)	14.6% allergic diseases (n = 6)	14.4% anaphylactic and anaphylactoid reactions (n = 104)	14.9% anaphylactic and anaphylactoid reactions (n = 41)
3.	9.1% allergic diseases (n = 2)	8.0% bullous diseases (n = 3)	12.8% allergic diseases (n = 5)	5.8% allergic diseases (n = 3)	14.6% general clinical signs and symptoms (n = 6)	11.8% urticaria (n = 84)	11.6% allergic diseases (n = 32)
4.	9.1% ocular diseases (n = 2)	8.0% swelling and edema of the soft tissues of the mouth (n = 2)	7.7% anaphylactic and anaphylactoid reactions (n = 3)	5.8% bullous reactions (n = 3)	9.8% anaphylactic and anaphylactoid reactions (n = 4)	9.7% allergic diseases (n = 69)	8.7% urticaria (n = 24)
5.	9.1% bullous diseases (n = 2) 9.1% urticaria (n = 2)		7.7% bullous reactions (n = 3)	3.8% general clinical signs and symptoms (n = 2)	9.8% swelling and edema of the soft tissues of the mouth (n = 4) 9.8% urticaria (n = 4)	7.2% dermatitis and eczema (n = 51)	7.3% bullous reactions (n = 20)

HLT = High Level Term (analysis level of MedDRA terminology). ^1^In 16.1% (n = 223) of the hypersensitivity reports the age of the patient was unknown. Therefore, not all hypersensitivity reports could be assigned to the defined age groups. ^2^Chronic obstructive pulmonary disease (COPD). ^3^Reported hypersensitivities and allergies in the patient history are pooled together since a clear separation into hypersensitivities and allergies is not possible due to the non-differentiating coding. ^4^The seriousness of the ADR report is based on the legal definition of the German Drug Law [30]. An ADR report is classified as serious if the reported ADR was serious or life-threatening, resulted in hospitalization or prolongation thereof, led to death, or resulted in permanent disability or a congenital anomaly. ^5^Shown are the five β-lactam antibiotics reported most frequently in each age group. One hypersensitivity report may contain more than one drug reported as suspected. Therefore, the total number of drugs reported exceeds the total number of hypersensitivity reports. ^6^Shown are the five most commonly reported types of hypersensitivity reactions at the HLT level of the MedDRA terminology [28]. One hypersensitivity report may contain multiple types of hypersensitivity reactions. These may be assigned to different higher-level codes. As a result, the total number of types of hypersensitivity reactions coded at the HLT level shown exceeds the total number of hypersensitivity reports. ^7^The HLT term „rashes, eruptions and exanthems“ cannot be differentiated further with regard to the type of rash. Therefore, no statements can be made as to whether the rashes were urticarial, in the sense of an immediate-type reaction, or maculo-papular, in the sense of a delayed-type reaction.

**Table 4.** The most frequently reported β-lactam antibiotics with their reporting rates, reported indications, routes of administration, and types of hypersensitivity reactions.

**Absolute and relative number of reports of the five β-lactam antibiotics most frequently reported as suspected/interacting, total number of coded β-lactam antibiotics** *^1^*	**Number of hypersensitivity reports per 1,000,000 outpatient prescriptions (Research Institute for Ambulatory Health Care in Germany)**	**The three most frequently reported indications (PT level) per coded β-lactam antibiotics** *^2^*	**The three most frequently reported routes of administration per coded β-lactam antibiotics** *^3^*	**Absolute and relative number of reports of the five most frequently reported types of hypersensitivity reactions (HLT level)** *^4^*
**Amoxicillin** 728 (52.5%), 749 – Serious: 34.9% (n = 254) – Death: 0.5% (n = 4) – Life-threatening: 3.4% (n = 25) – Hospitalization: 16.1% (n = 117)	Total: 13.8 Age strata: – 0 – 1 years: 7.6 – 2 – 3 years: 2 – 9 – 4 – 6 years: 5.9 – 7 – 12 years: 6.4 – 13 – 18 years: 7.1 – 19 – 65 years: 14.7 – ≥ 66 years: 15.7	– 4.3% bronchitis (n = 32) – 4.1% tonsillitis (n = 31) – 4.1% sinusitis (n = 24) – 22.3% no information (n = 167)	– 69.7% oral (n = 522) – 0.3% transmammary (n = 2) – 0.3% IV (n = 2) – 29.5% no information (n = 221)	– 56.9% rashes, eruptions, and exanthems (n = 414) – 12.5% urticaria (n = 91) – 10.4% allergic diseases (n = 76) – 6.6% dermatitis and exanthema (n = 48) – 6.0% dermatitis caused by specific agent (n = 44)
**Cefuroxime** 267 (19.3%), 290 – Serious: 73.8% (n = 197) – Death: 4.9% (n = 13) – Life-threatening: 30.0% (n = 80) – Hospitalization: 37.8% (n = 101)	Total: 6.9 Age strata: – 0 – 1 years: 0.0 – 2 – 3 years: 3.1 – 4 – 6 years: 3.6 – 7 – 12 years: 3.4 – 13 – 18 years: 3.9 – 19 – 65 years: 6.0 – ≥ 66 years: 6.5	– 20.3% antibiotic prophylaxis (n = 59) – 6.2% sinusitis – (n = 18) – 5.5% bronchitis – (n = 16) – 28.3% no information (n = 82)	– 41.4% oral (n = 120) – 32.4% IV (n = 94) – 5.2% ophthalmic/ intraocular (n = 15) – 20.7% no information (n = 60)	– 39.0% anaphylactic and anaphylactoid reactions (n =104) – 22.1% rashes, eruptions and exanthems (n = 59) – 9.7% allergic diseases (n = 26) – 7.1% urticaria (n = 19) – 6.4% circulatory collapse and shock (n = 17)
**Ampicillin/sulbactam** 80 (5.8%), 92 – Serious: 56.3% (n = 45) – Death: 1.3% (n = 1) – Life-threatening: 3.8% (n = 3) – Hospitalization: 37.5% (n = 30)	Total: 296.6 Age strata: – 0 – 1 years: 0.0 – 2 – 3 years: 0.0 – 4 – 6 years: 0.0 – 7 – 12 years: 0.0 – 13 – 18 years: 145.4 – 19 – 65 years: 270.6 – ≥ 66 years: 325.6	– 10.9% pneumonia (n = 10) – 5.4% erysipelas (n = 5) – 5.4% urinary tract infection (n = 5) – 20.7% no information (n = 19)	– 40.2% oral (n = 37) – 29.3% IV (n = 27) – 30.5% no information (n = 27)	– 52.5% rashes, eruptions, and exanthems (n =42) – 10.0% bullous reactions (n = 8) – 8.8% allergic diseases (n = 7) – 8.8% dermatitis caused by specific agent (n = 7) – 7.5% allergies to food, food additives, drugs, and other chemicals (n = 6)
**Cefaclor** 55 (4.0%), 62 – Serious: 54.5% (n = 30) – Death: 0.0% (n = 0) – Life-threatening: 5.5% (n = 3) – Hospitalization: 21.8% (n = 12)	Total: 3.8 Age strata: – 0 – 1 years: 3.7 – 2 – 3 years: 4.0 – 4 – 6 years: 2.8 – 7 – 12 years: 3.2 – 13 – 18 years: 1.0 – 19 – 65 years: 2.3 – ≥ 66 years: 1.9	– 11.3% bronchitis (n = 7) – 8.1% otitis media (n = 5) – 8.1% tonsillitis (n = 5) – 21.0% no information (n = 13)	– 67.7% oral (n = 42) – 32.3% no information (n = 20)	– 49.1% rashes, eruptions and exanthems (n = 27) – 34.5% urticaria (n = 19) – 14.5% allergic diseases (n = 8) – 7.3% dermatitis and exanthema (n = 4) – 7.3% general clinical signs and symptoms (n = 4)
**Phenoxymethylpenicillin** 52 (3.7%), 55 – Serious: 48.1% (n = 25) – Death: 0.0% (n = 0) – Life-threatening: 1.9% (n = 1) – Hospitalization: 15.4% (n = 8)	Total: 3.1 Age strata: – 0 – 1 years: 4.5 – 2 – 3 years: 1.9 – 4 – 6 years: 1.4 – 7 – 12 years: 3.2 – 13 – 18 years: 0.7 – 19 – 65 years: 3.0 – ≥ 66 years: 6.5	– 12.7% tonsillitis (n = 7) – 10.9% erysipelas (n = 6) – 9.1% scarlet fever (n = 5) – 23.6% no information (n = 13)	– 74.5% oral (n = 41) – 3.6% IV (n = 2) – 21.8% no information (n = 11)	– 46.2% rashes, eruptions, and exanthems (n = 24) – 19.2% urticaria (n = 10) – 11.5% allergic diseases (n = 6) – 5.8% angioedema (n = 3) – 5.8% general clinical signs and symptoms (n = 3)

PT = Preferred Term (analysis level of MedDRA terminology), HLT = High Level Term (analysis level of MedDRA terminology). ^1^The total number of coded β-lactam antibiotics may differ from the total number of hypersensitivity reports per β-lactam antibiotic. This may occur, e.g., if more than one treatment cycle per ADR report is described, or if the route of administration or the indication of the β-lactam antibiotic was changed. Therefore, the total number of coded β-lactam antibiotics may exceed the total number of reports for the respective β-lactam antibiotic. ^2^Shown are the three most frequently reported indications at preferred term level of MedDRA terminology[23]. The relative share of indications is related to the total number of coded β-lactam antibiotics. ^3^Shown are the three most frequently reported routes of administration. The relative share of the routes of administration is related to the total number of coded β-lactam antibiotics. ^4^Shown are the five most common reported types of hypersensitivity reactions at the HLT level of MedDRA terminology [23]. One hypersensitivity report may report multiple types of hypersensitivity reactions. As a result, the total number of types of hypersensitivity reactions exceeds the total number of hypersensitivity reports. ^5^The HLT term „rashes, eruptions, and exanthems” cannot be differentiated further with regard to the type of rash. Therefore, no statements can be made as to whether the rashes were urticarial, in the sense of an immediate-type reaction, or maculo-papular, in the sense of a delayed-type reaction.

**Table 5.** Stratified analysis of anaphylactic and bullous reactions. Stratified analysis of reports referring to anaphylactic and bullous reactions with regard to the demographic parameters and histories of the patients, the seriousness criteria of the hypersensitivity reports, the β-lactam antibiotics most frequently reported, and their primary reporting sources.

	**Reports reporting anaphylactic reactions (n = 182)**	**Reports not reporting anaphylactic reactions (n = 1,205)**	**OR [+/– adj. 95 CI] reports anaphylactic reactions versus no anaphylactic reactions**	**Reports reporting bullous reactions (n = 68).**	**Reports not reporting bullous reactions (n = 1,319)**	**OR [+/– adj. 95 CI] reports bullous reactions versus no bullous reactions**
Demographic parameters of the patients						
Average age of patients (median) [in years]^1^	53.6 (56.5)	45.6 (50)		48.9 (50.0)	46.5 (50)	
Female	56.6% (n = 103)	59.0% (n = 711)	1.0 [0.6 – 1.6]	52.9% (n = 36)	59.0% (n = 778)	0.8 [0.4 – 1.7]
Male	39.0% (n = 71)	38.8% (n = 468)		44.1% (n = 30)	38.6% (n = 509)	
Sex unknown	4.4% (n = 8)	2.2% (n = 26)		2.9% (n = 2)	2.4% (n = 32)	
Patient histories						
Hypertension	17.0 (n = 31)	7.6% (n = 92)	2.5 [1.0 – 6.1]	16.2% (n = 11)	8.5 (n = 112)	2.1 [0.7 – 5.9]
Cardiovascular diseases	10.4% (n = 19)	3.8% (n = 46)	2.9 [0.9 – 9.7]	11.8% (n = 8)	4.3% (n = 57)	3.0 [0.9 – 10.0]
Diabetes	9.3% (n = 17)	4.0% (n = 48)	2.5 [0.9 – 9.5]	10.3% (n = 7)	4.4% (n = 58)	2.5 [0.7 – 9.0]
Asthma	6.6% (n = 12)	1.5% (n = 18)	4.7 [0.9 – 23.1]	0.0% (n = 0)	2.2% (n = 30)	–
COPD^2^	6.0% (n = 11)	1.3% (n = 16)	4.8 [0.9 – 25.7]	5.9% (n = 4)	1.7% (n = 23)	3.5 [0.6 – 19.1]
Hypersensitivities/allergies^3^	19.8% (n = 36)	13.3% (n = 160)	1.6 [0.7 – 3.6]	14.7% (n = 10)	14.1% (n = 186)	1.1 [0.4 – 3.1]
Seriousness of hypersensitivity reports^4^						
Serious	99.5% (n = 181)	44.0% (n = 531)	229.7 [158.7 – 332.5]	86.8% (n = 59)	49.5% (n = 653)	6.7 [2.2 – 20.1]
Death	9.3% (n = 17)	1.9% (n = 23)	5.3 [1.3 – 20.9]	29.5% (n = 20)	1.5% (n = 20)	27.1 [9.4 – 78.1]
Life-threatening	53.8% (n = 98)	5.0% (n = 60)	22.3 [11.0 – 45.0]	19.1% (n = 13)	11.0% (n = 145)	1.9 [0.7 – 5.1]
Hospitalization	53.8% (n = 98)	20.0% (n = 241)	4,7 [2.8 – 7,8]	52.9% (n = 36)	23.0% (n = 303)	3.8 [1.8 – 8.1]
Disabling	2.7% (n = 5)	1.2% (n = 15)	2.2 [0.2 – 20.6]	2.9% (n = 2)	1.4% (n = 18)	2.2 [0.2 – 218]
Five most commonly reported β-lactam antibiotics in anaphylactic and bullous reaction reports, respectively^5^						
1.	cefuroxime (n = 104)	cefuroxime (n = 162)	8.6.[5.0 – 14.7]	amoxicillin (n = 20)	amoxicillin (n = 695)	0.4 [0.2 – 0.9]
2.	amoxicillin (n = 28)	amoxicillin (n = 687)	0.1 [0.1 – 0.3]	cefuroxime (n = 14)	cefuroxime (n = 252)	1.1 [0.4 – 2.8]
3.	cefazolin (n = 19)	cefazolin (n = 9)	15.5 [2.7 – 88.1]	piperacillin (n = 9)	piperacillin (n = 26)	7.6 [2.2 – 26.3]
4.	piperacillin (n = 8)	piperacillin (n = 27)	2.0 [0.4 – 11.3]	ampicillin/sulbactam (n = 7)	ampicillin/sulbactam (n = 68)	2.1 [0.6 – 7.5]
5.	ceftriaxone (n = 7)	ceftriaxone (n = 36)	1.3 [0.2 – 7.7]	ceftazidime (n = 5)	ceftazidime (n = 4)	26.1 [3.3 – 207.9]
Primary reporting source of the hypersensitivity reports^6^						
Physician	51,1% (n = 93)	41.7% (n = 503)	1.5 [0.9 – 2.4]	47.1% (n = 32)	42.8% (n = 564)	1.2 [0.6 – 2.5]
Pharmacist	15.9% (n = 29)	17.8% (n = 215)	0.9 [0.4 – 2.0]	8.8% (n = 6)	18.0% (n = 238)	0.4 [0.1 – 1.6]
Patient	11.0% (n = 20)	29.0% (n = 349)	0.3 [0.1 – 0.8]	16.2% (n = 11)	27.1% (n = 358)	0.5 [0.2 – 1.4]

^1^In 16.1% (n = 223) of hypersensitivity reports, the age of the patient was unknown. ^2^Chronic obstructive pulmonary disease (COPD). ^3^Reported hypersensitivities and allergies in the patient history are pooled together since a clear separation into hypersensitivities and allergies is not possible due to the non-differentiating coding. ^4^The seriousness of the ADR report is based on the legal definition of the German Drug Law [30]. An ADR report is classified as serious if the reported ADR was serious or life-threatening, resulted in hospitalization or prolongation thereof, led to death, or resulted in permanent disability or a congenital anomaly. ^5^Shown are the five β-lactam antibiotics most frequently reported in the reports of anaphylactic and bullous reactions, respectively. ^6^Only reports referring to a single primary reporting source are shown. For example, reports which were reported by a physician and a patient are not considered in this analysis.

## Supplemental material

Supplemental Figure 1.
