# Supplementary material for: Descriptive analysis of adverse drug reaction reports for hypersensitivity reactions stratified in relation to different beta-lactam antibiotics
Source: Allergol Select. 2022 Feb 3;6:42–60. doi: 10.5414/ALX02189E (PMC8822522; doi:10.5414/ALX02189E)
Supplement: Supplemental Figure 1. [file allergologieselect-6-042-S01.pdf]

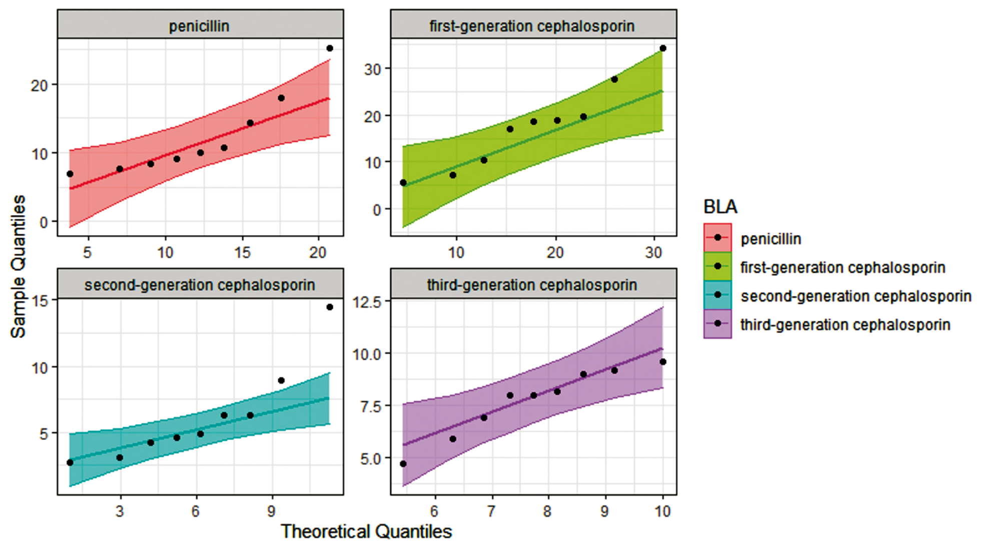
penicillin
first-generation cephalosporin
second-generation cephalosporin
third-generation cephalosporin
Sample Quantiles
Theoretical Quantiles
BLA
penicillin
first-generation cephalosporin
second-generation cephalosporin
third-generation cephalosporin
